# Decoupling the impact of bulk and surface point defects on the photoelectrochemical properties of LaFeO_3_ thin films†

**DOI:** 10.1039/d2sc04675j

**Published:** 2022-09-06

**Authors:** Xin Sun, Devendra Tiwari, Meicheng Li, David J. Fermin

**Affiliations:** State Key Laboratory of Alternate Electrical Power System with Renewable Energy Sources, School of New Energy, North China Electric Power University Beijing 102206 China mcli@ncepu.edu.cn; Department of Mathematics, Physics and Electrical Engineering, Northumbria University Ellison Building Newcastle Upon Tyne NE1 8ST UK; School of Chemistry, University of Bristol Cantocks Close, Bristol BS8 1TS UK david.fermin@bristol.ac.uk

## Abstract

Point defects (PDs) play a key role in the properties of semiconductor photoelectrodes, from doping density to carrier mobility and lifetime. Although this issue has been extensively investigated in the context of photovoltaic absorbers, the role of PDs in photoelectrodes for solar fuels remains poorly understood. In perovskite oxides such as LaFeO_3_ (LFO), PDs can be tuned by changing the cation ratio, cation substitution and oxygen content. In this paper, we report the first study on the impact of bulk and surface PDs on the photoelectrochemical properties of LFO thin films. We independently varied the La : Fe ratio, within 10% of the stoichiometric value, in the bulk and at the surface by tuning the precursor composition as well as selective acid etching. The structure and composition of thin films deposited by sol–gel methods were investigated by SEM-EDX, ICP-OES, XPS and XRD. Our analysis shows a correlation between the binding energies of Fe 2p_3/2_ and O 1s, establishing a link between the oxidation state of Fe and the covalency of the Fe–O bond. Electrochemical studies reveal the emergence of electronic states close to the valence band edge with decreasing bulk Fe content. DFT calculations confirm that Fe vacancies generate states located near the valence band, which act as hole-traps and recombination sites under illumination. Dynamic photocurrent responses associated with oxygen reduction and hydrogen evolution show that the stoichiometric La : Fe ratio provides the most photoactive oxide; however, this can only be achieved by independently tuning the bulk and surface compositions of the oxide.

## Abstract 2

This article shows, for the first time, the delicate balance between controlling bulk recombination sites and dynamics of interfacial carrier transfer *via* independently tuning the bulk and surface compositions of perovskite oxide thin films.

## Graphical abstract

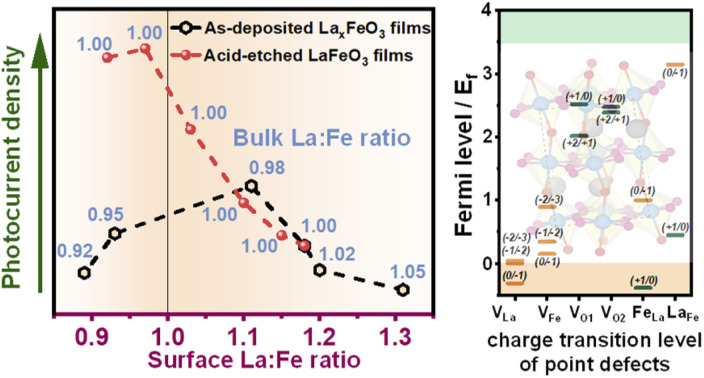

## Introduction

Metal oxide solar absorbers have been widely investigated in the context of photoelectrochemical (PEC) solar fuel generation and photocatalysis due to their low-cost, scalable processing and photostability in aqueous electrolytes.^1–3^ Lanthanum ferrite, LaFeO_3_ (LFO), is an interesting p-type ferrite with band gap values reported in the range of 2.1 to 2.7 eV and band edge energies straddling both water oxidation and reduction potentials. Several studies have shown that LFO can achieve overall water splitting in the visible range, although significant differences have been observed depending on preparation methods.^4–9^ For instance, highly crystalline LFO nanoparticles obtained by calcination at temperatures above 700 °C show photovoltages for the hydrogen evolution reaction as high as 1.4 *vs.* RHE but low quantum yield,^8,10^ while thin-film electrodes prepared at lower temperatures are characterised by a high degree of interfacial recombination.^4,5,9^ Efforts towards increasing the external quantum efficiency (EQE) of LFO include partial cation substitution,^9,11,12^ heterostructure design,^10,13^ and preparation methods.^14^

The poor mobility of charge carriers in LFO is a key parameter in photoelectrode performance, contributing to not only losses of minority carriers by surface and bulk recombination losses but also losses of majority carriers through water oxidation. Indeed, Celorrio *et al.* demonstrated that the valence band edge of nanostructured LFO is slightly above the water redox potential, thus water oxidation can compete with hole collection at the back contact.^8^ We have demonstrated a significant increase in photocurrent responses by depositing a thin overlayer of TiO_2_ onto LFO nanoparticles, which acts as a selective hole-barrier at the semiconductor/electrolyte interface.^10^ A potential bulk defect mitigation strategy in this class of ternary metal oxides involves tuning the cation ratio. Lee and co-workers reported the photocurrent density of epitaxial BiVO_4_ photoanodes to obtain considerable changes upon the tuning of the surface Bi : V ratio.^15^ We have recently demonstrated that photoanodic responses in n-type GaFeO_3_ thin films are significantly affected by the Ga : Fe ratio.^16^ Tuning the cation ratio leads to point defects (PDs), generating states that can change the carrier concentration, trap states and even states that can facilitate minority carrier transfer. There is a profound knowledge gap on the role of PDs in the photoelectrochemical properties of compound semiconductors.

In this work, we explore the composition dependence of photoresponses of LFO thin-films obtained by sol–gel methods over a small range around the La/Fe stoichiometric ratio. Composition analysis by ICP-OES and EDX, along with XRD analysis, shows that a single orthorhombic LFO phase can be obtained in the range of bulk La : Fe ratios between 0.92 and 1.09. Electrochemical studies conducted under alkaline conditions show the increase of density of states near the valence band edge of LFO with an increased La : Fe ratio. These states act as bulk recombination centres, decreasing the photoelectrochemical responses in La-rich films. Furthermore, carefully conducted acid etching of LFO films allowed tuning the surface La : Fe ratio while keeping the bulk composition constant. We correlated DFT electronic structure calculations and PD energy formation with electrochemical measurements, uncovering, for the first time, the impact on the photoelectrode performance of PDs such as Fe vacancies in the bulk and at the surface.

## Experimental and computational methodology

### Thin-film preparation

Near stoichiometric LFO thin films were prepared *via* sol–gel routes composed of 1 mL of 0.5 M La(NO_3_)_3_·6H_2_O ethanolic solution, 1 mL of 0.5 M Fe(NO_3_)_3_·9H_2_O ethanolic solution and 1 mL of 1 M citric acid ethanolic solution mixed in a capped glass vial containing 0.1 mL ultrapure Milli-Q water (18.25 MΩ).^9^ After 2 h stirring the precursor solution, 0.125 mL of ethylene glycol was dropped into the clear solution, and the mixture was further stirred for 20 h at room temperature. The as-prepared solution is deposited onto either glass slides (XRD measurements) or F-doped SnO_2_ coated glass (FTO) by spin-coating at 3000 rpm for 30 s. The wet film is dried at 100 °C for 10 min and heated at 400 °C for 1 h. The spin-coating step was repeated three times to achieve a film thickness of approximately 130 nm. Finally, the LFO films were calcined at 600 °C for 3 h to increase the crystallinity. The content of ferric nitrate and lanthanum nitrate added into the precursor solution is adjusted to prepare L_*x*_FO, where *x* is the La : Fe molar ratio in the precursor solution. Table S1 in the ESI† shows the bulk La : Fe ratio of the thin-films measured by ICP-OES and EDX, confirming that the nominal ratio in the sol–gel precursor (*x*) closely reflects the film composition. In order to vary the surface La : Fe ratio, near stoichiometric LFO films were etched in nitric acid solution pH 1. These samples are labelled LFO-*y*, where *y* is the acid-etching time going from 10 to 40 s. Details on characterisation tools and photoelectrochemical measurements are described in the ESI†

### Computational methods

DFT calculations were performed using the CASTEP 18.1 code package. A dense Monkhorst–Pack *k*-point grid with spacing <0.35 Å^−1^ was implemented in all calculations, along with norm-conserving pseudopotentials with an energy cut-off of 1100 eV (∼81 Ry). Strict convergence tolerances of 1 neV and 1 meV Å^−1^ were implemented for electronic and ionic convergences, respectively. The geometry was optimised using GGA+U formalism in a spin-polarised scheme using the PBESol functional and Hubbard *U*_effective_ on Fe 3d states of 4.7 eV and BFGS minimisation. A linear response approach to calculation of *U*_effective_ for Fe in 150 compounds reported in the Materials Project database has resulted in values averaging at 4.76 ± 0.79.^17,18^ For the current system, therefore, the optimisation of *U*_effective_ for Fe 3d states was done by empirically varying this parameter between 3.9 and 5.1 eV in intervals of 0.2 eV and comparing the calculated structural parameters and magnetic moments with experimental results. The optimised value of 4.7 eV was found to yield a structure closest to the experimentally determined structure from high-resolution powder neutron diffraction,^19^ and a magnetic moment of 4.6 *μ*_B_ similar to previous experiments.^20^ As described in Table S2,† the lattice parameters of the optimised Pbnm orthorhombic unit cell are within 1% of room temperature experimental values reported for stoichiometrically pure LaFeO_3_,^19^ while bond-angles, bond-lengths and FeO_6_ octahedral tilt angles also close to experimental values. The density of states was calculated using the HSE06 hybrid functional on the structure relaxed using DFT+U implementation.

## Results and discussion

**Fig. 1:**
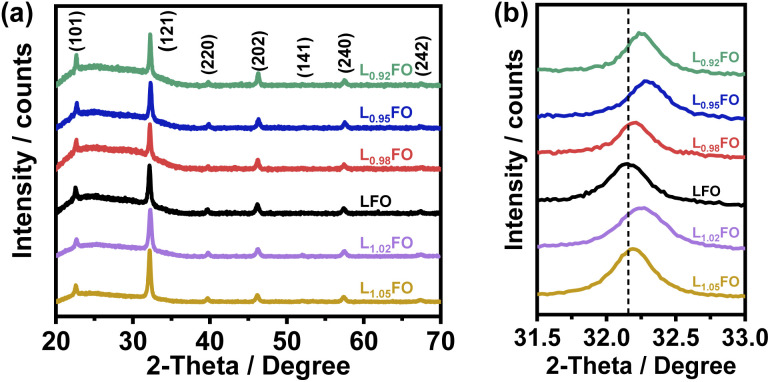
(a) XRD patterns of various LFO thin films and (b) evolution of the (121) peak as a function of the bulk La : Fe ratio. The dashed line marks the peak position of stoichiometric LFO thin films. Diffraction patterns across the composition range correspond to high phase pure orthorhombic LFO with a *Pbnm* space group.

Fig. 1a shows the X-ray diffraction (XRD) patterns of L_*x*_FO thin films with various La : Fe ratios (*x*) deposited onto glass slides, which are all consistent with a pure orthorhombic phase (Pbnm space group) in agreement with previous reports.^9^ L_*x*_FO films deposited either onto glass slides or FTO show similar morphology with a thickness of 130 nm after annealing at 600 °C. As shown in Fig. S1 of the ESI,† acid etching of the LFO film does not affect the XRD pattern. Fig. 1b shows that the (121) diffraction peak for the La-poor L_*x*_FO is slightly shifted toward higher angles in comparison to near stoichiometric cation composition (*x* = 1). This observation may suggest the promotion of La vacancies which lead to a higher Fe oxidation state and a decrease of the lattice constant, primarily in films with *x* < 1. The behaviour observed in La-rich films is more complex and will be discussed further below.

**Fig. 2:**
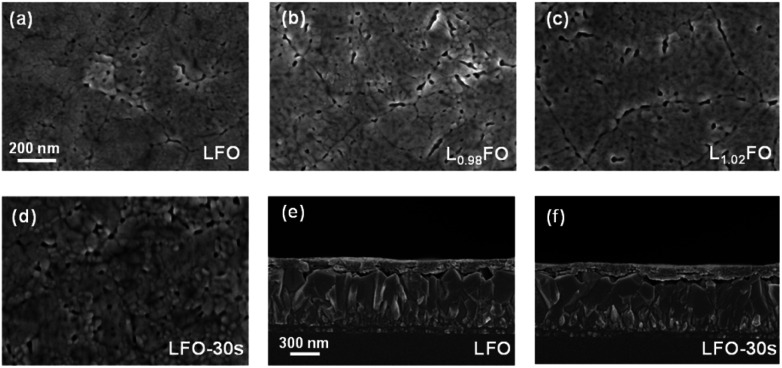
Scanning electron microscopy (SEM) top view images of LFO thin films with different *x* values (a–c) and of LFO after 30s nitric acid etching (d). Cross-sectional SEM images of LFO before (e) and after (f) acid etching show that the film thickness (130 nm) and integrity are not affected.

Fig. 2a–c show that the surface morphologies of L_*x*_FO films with different La : Fe ratios are similar. The films are characterised by compact nanoscale grains and a low corrugation. The acid etching does not significantly affect the topography and grain size of the LFO thin film (Fig. 2d). Another key observation comes from the cross-sectional views in Fig. 2e and f of the LFO films prior to and after 30s acid etching. These results confirm that the integrity of the 130 nm film is not affected by the acid treatment. However, we do observe that longer etching times lead to substantial material dissolution.

**Fig. 3:**
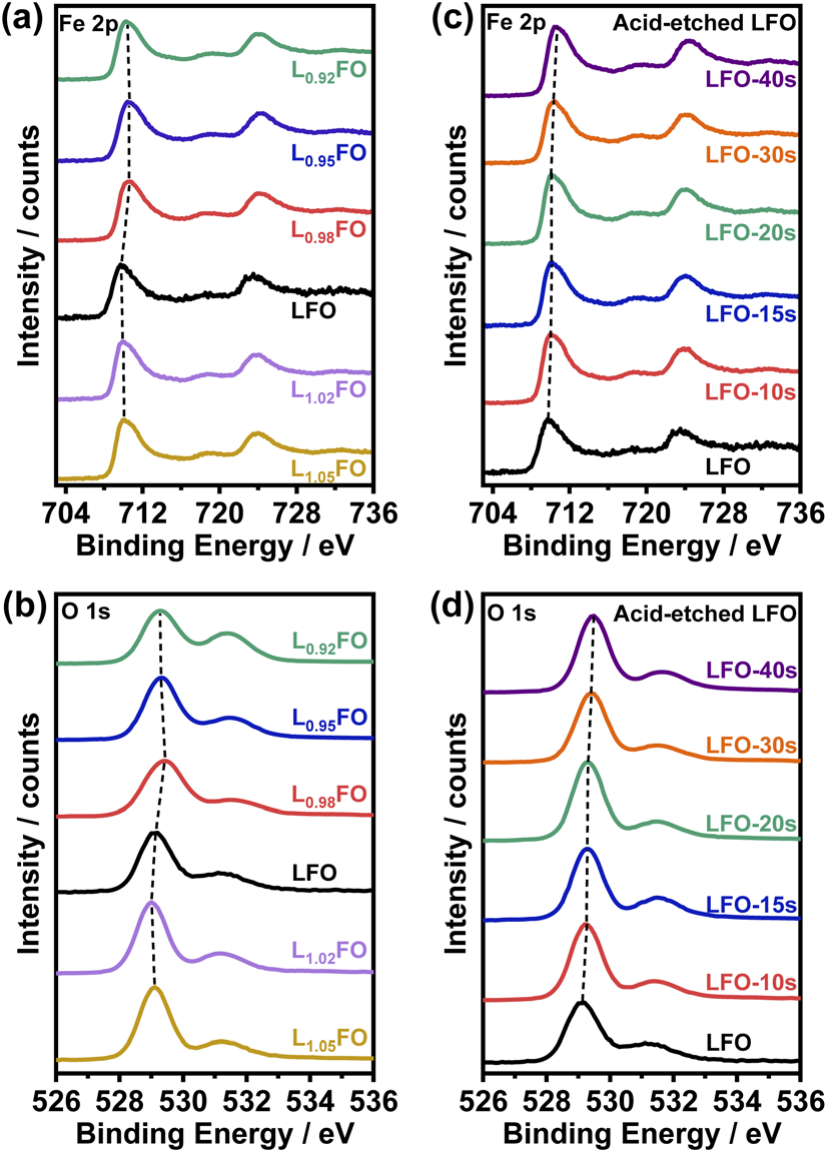
Fe 2p (a) and O 1s (b) photoemission spectra of L_*x*_FO thin films with various bulk cation ratios. These photoemission lines are also shown in LFO films (*x* = 1) etched in nitric acid for up to 40 s (c, d).

XPS spectra of LFO thin films as a function for the various cation ratios are illustrated in Fig. 3. Photoemission in the Fe 2p region (Fig. 3a and c) shows contributions from Fe 2p_3/2_ and Fe 2p_1/2_ around 710 eV and 724 eV, respectively, as well as satellite peaks at +8.3 eV from these two main peaks. The binding energies are consistent with Fe in the +3 oxidation state.^9,16^ The O 1s photoemission line shows the contribution from several species (Fig. 3b and d), with the most prominent centred at 529 eV corresponding to oxygen in the perovskite lattice, while the higher binding energy peaks are associated with hydroxyl, carbonates and adsorbed water.^21^ Table S3† compiles the surface La : Fe ratios obtained by integrating the Fe 2p and La 4d (not shown) photoemission spectra as a function of the bulk cation ratio and the acid etching time. The data show that only when the bulk ratio is below 0.95 the LFO surface is Fe-rich. On the other hand, analysis of the acid-etched samples confirms that diluted nitric acid selectively etches La from the surface. Selective La etching in perovskite oxides has been reported previously,^22^ which has been rationalised in terms of the larger La–O bond length and higher surface energy in comparison to the B-site cation.^23^

**Fig. 4:**
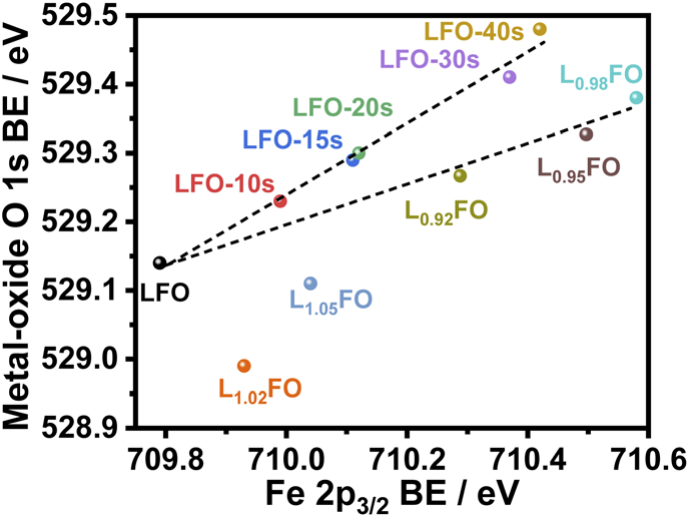
Correlation between Fe 2p_3/2_ and metal-oxide O 1s binding energy (BE) in thin films with different La : Fe ratios and after increasing acid etching time.

Fig. 4 illustrates the complex correlation between the lattice O 1s and Fe 2p_3/2_ binding energies in L_*x*_FO and acid-etched LFO. As described in our previous work, the Fe 2p_3/2_ binding energy is linked to the surface oxidation state, while the position of O 1s reflects the Fe–O bond covalency.^9^ The trend in the acid-etched samples shows a clear scaling relation between these two binding energies, suggesting that decreasing La content (increasing etching time) increases the oxidation state of Fe, increasing the bond covalency. Indeed, increasing Fe oxidation state decreases the ionic radius and the Fe–O bond length.^9^ On the other hand, a significant deviation from linearity is observed upon changes in the bulk La : Fe ratio in the region of La-rich films. This trend suggests a complex interplay between Fe vacancies (V_Fe_) and other PDs, including oxygen vacancies (V_O_) as described in other studies.^24^ Furthermore, these observations are consistent with the complex trend in the XRD patterns shown in Fig. 1; thus, we have two independent measurements confirming a non-monotonic change in lattice constants upon changes in the La : Fe ratio. These defect states do not clearly manifest themselves in the absorptance spectra of the LFO films, as shown in Fig. S2.† As estimated from Tauc plots, the optical band gap is approximately 2.7 eV for all compositions.

**Fig. 5:**
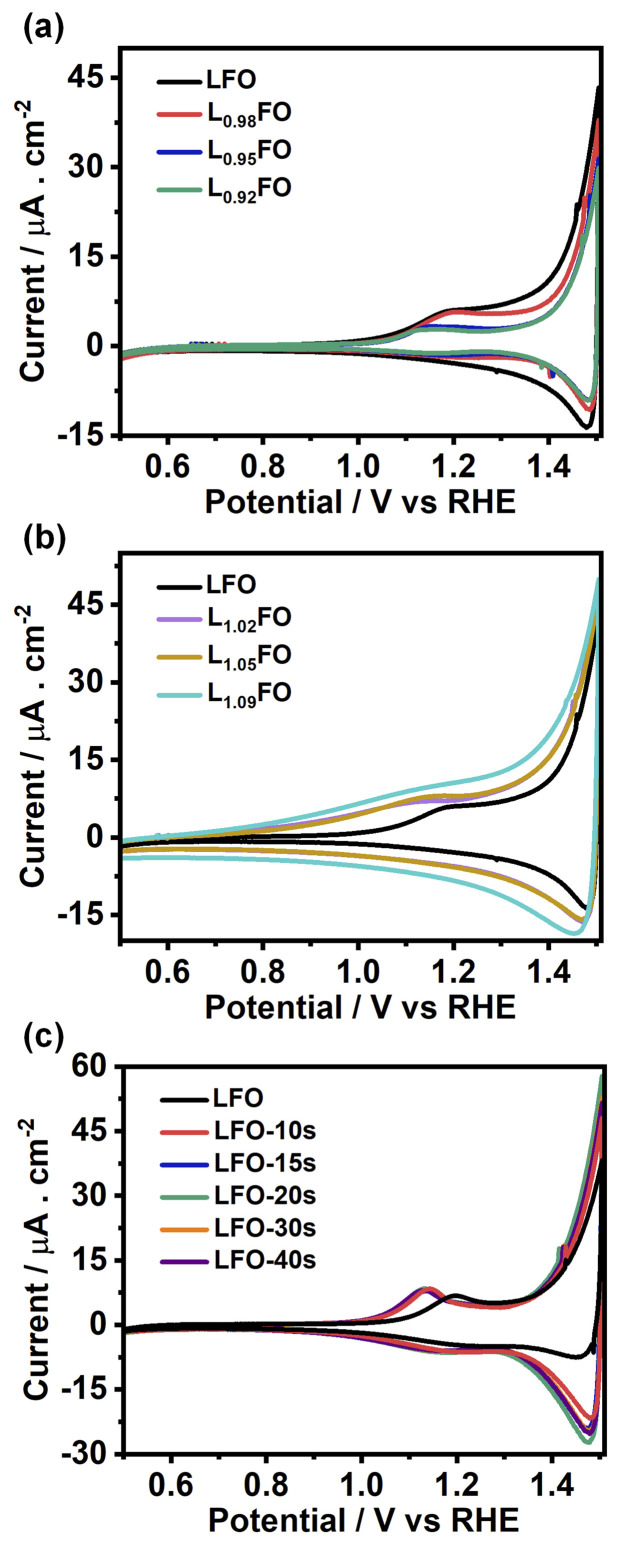
Cyclic voltammograms of 130 nm LFO thin films conducted at 50 mV s^−1^ in O_2_-saturated 0.1 M Na_2_SO_4_ aqueous solution at pH 12, as a function of the La : Fe ratio (a, b) and after various acid etching times (c). A systematic increase in the capacitive current in the range of 0.7 to 1.4 V is observed as the La : Fe ratio increases, revealing the presence of electronic states close to the valence band edge.

Fig. 5a and b show the cyclic voltammograms (CVs) of various LFO thin films with different La : Fe ratios recorded at 50 mV s^−1^ in O_2_-saturated 0.1 M Na_2_SO_4_ aqueous solution at pH 12 in the dark. A sharp increase in the current at potentials more positive than 1.4 V suggests a transition from depletion to accumulation. Capacitance potential curves (Fig. S3a†) measured at 1 kHz show a sharp increase at potential above 1.3 V in La-poor films, which systematically shifts to more positive potentials as the La content increases. This behaviour manifests itself in Mott–Schottky plots (Fig. S3b†) as an apparent shift of the flat band potential towards more positive values as well as an increase of acceptor density with increasing La content. However, these measurements should be considered with caution, given the contribution of defect-related states near the valence band. Indeed, the broadening of the capacitance responses between 1.0 V and 1.3 V *vs.* RHE corresponds to the population/depopulation of defect states.^25^ Our previous work demonstrated that cation disorder in LFO thin films could lead to forming intrinsic defect states close to the valence band edge.^9^ More importantly, we see a substantial broadening and increase in the charging current associated with these defects as the La-content increases, strongly suggesting that these states originate from Fe vacancies. In the case of acid-etched LFO (Fig. 5c), a sharpening of the voltammetric responses is observed even after short etching times, which also suggests a slight shift of the flat band potential towards slightly more negative values. However, no difference in the magnitude of capacitive responses (DOS) is observed, confirming that these states are related to bulk defects.

**Fig. 6:**
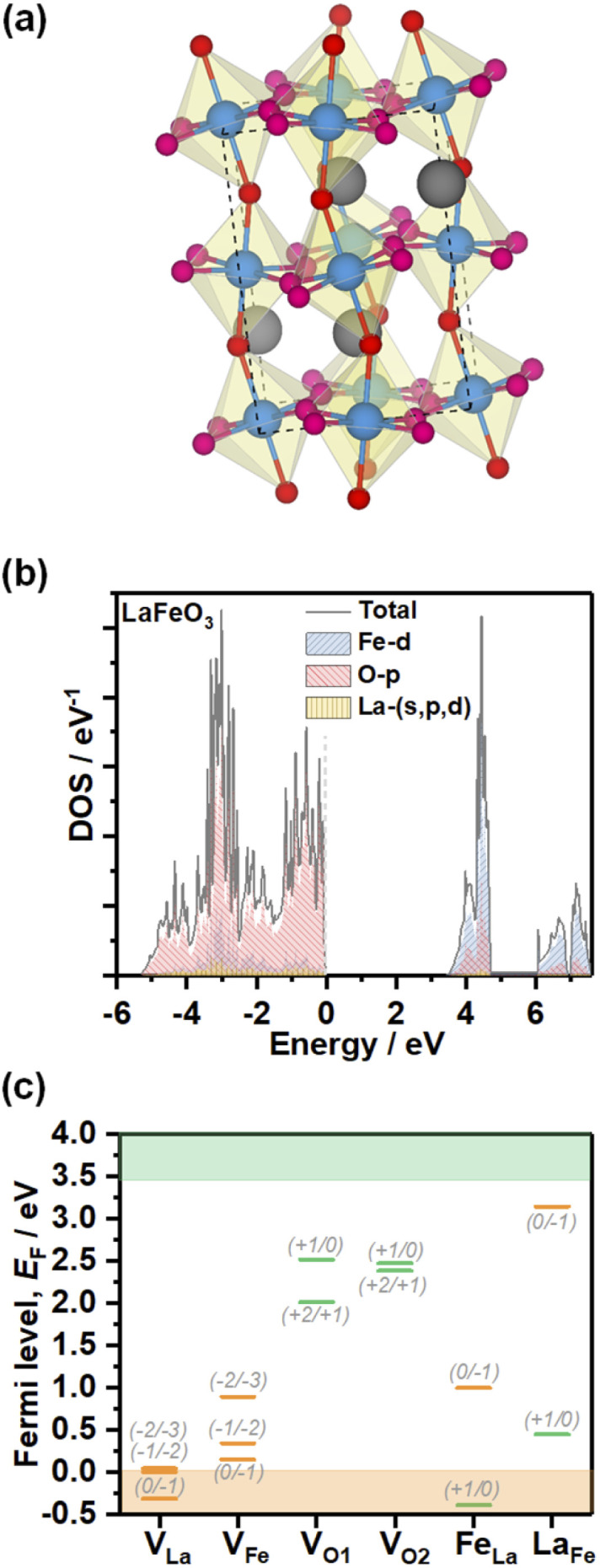
Electronic structure and PD energy calculations. Representation of the orthorhombic stoichiometric LFO (a). Projected density of states calculations obtained from the HSE06 hybrid functional (b). Charge transition levels of La, Fe and O vacancies (V_La_, V_Fe_, V_O1_ and V_O2_, respectively), as well as La_Fe_ and Fe_La_ antisite defects (c). Orange and green bars are used to designate acceptor and donor defect sites. Defect formation energy calculations were performed using DFT+U (Table S4†), while the charge transition levels were scaled to the band gap obtained from the hybrid HSE06 functional for consistency purposes.

In order to gain further understanding of the nature of defect states observed in electrochemical experiments, we performed DFT calculations of the electronic structure and PD energy formation. The element projected DOS calculations employing the HSE06 hybrid functional of stoichiometric LFO (*Pbnm*, unit cell illustrated in Fig. 6a) are shown in Fig. 6b. As expected, the valence band maximum (VBM) of this ionic material is primarily composed of the O 2p states with minimal contribution from Fe 3d states. The conduction band minimum is composed of Fe 3d states split into lower t_2g_ and higher e_g_ levels with a splitting of between 1 and 1.5 eV. Thus, the dominant optical LFO band gap is associated with a O 2p to Fe 3d charge-transfer transition even though the fundamental bandgap is an indirect transition. The calculated band gap from hybrid HSE06 functional implementation is 3.43 eV, larger than the 2.7 eV obtained experimentally. The HSE06 functional includes a fraction of exact Hartree–Fock (HF) exchange which neglects instantaneous electron–electron correlations, resulting in wider band gaps, especially for oxides.^26^ Within the HF formalism, for an N-electron system in the ground state, an electron experiences potential of only N-1 other electrons in occupied states compared to the potential felt in unoccupied states due to N electrons in the occupied states. Thus, an electron in an occupied state experiences more attractive ionic potential due to relatively less screening offered by one less electron, leading to lowering of valence band energy and widening of the band gap. However, features such as composition and widths of the bands in our calculations are consistent with XPS, XES and XAS analyses of epitaxial thin films grown by pulsed laser deposition,^27^ and bulk crystals prepared by solid-state reactions.^28^

Fig. 6c shows the charge transition level of various PDs obtained using DFT+U calculations as summarised in Table S4.† The thermodynamic defect charge transition level, *ε*(*D*,*q*/*q*′), is defined as the point at which the formation energies (*E*^*D*,*q*^) of defects in charge states *q* and *q*′ are equal, as described in eqn (1),1
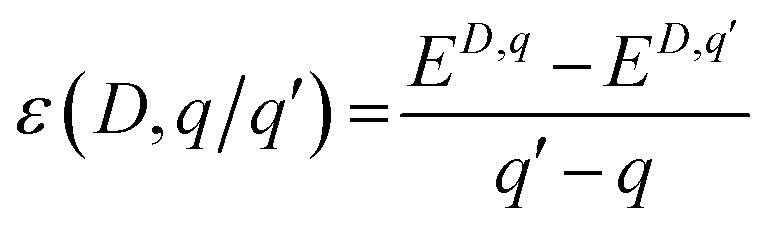


The analysis clearly shows that V_La_ and V_Fe_ generate defect states near the valence band edge, consistent with our voltammetric analysis. V_O1_ and V_O2_ generate deeper states which are located outside the electrochemical potential window. The states can have a profound effect on non-radiative bulk recombination losses. Fe_La_ and La_Fe_ antisite defects also generate deep states, although closer to the band edges than oxygen vacancies. It should be mentioned that Taylor and co-workers have explored a wider range of structural defects which can be present at different temperatures and oxygen contents.^24^ In any case, the relative positions of cation and oxygen vacancies with respect to the band edges do confirm the p-type conductivity of these materials.

**Fig. 7:**
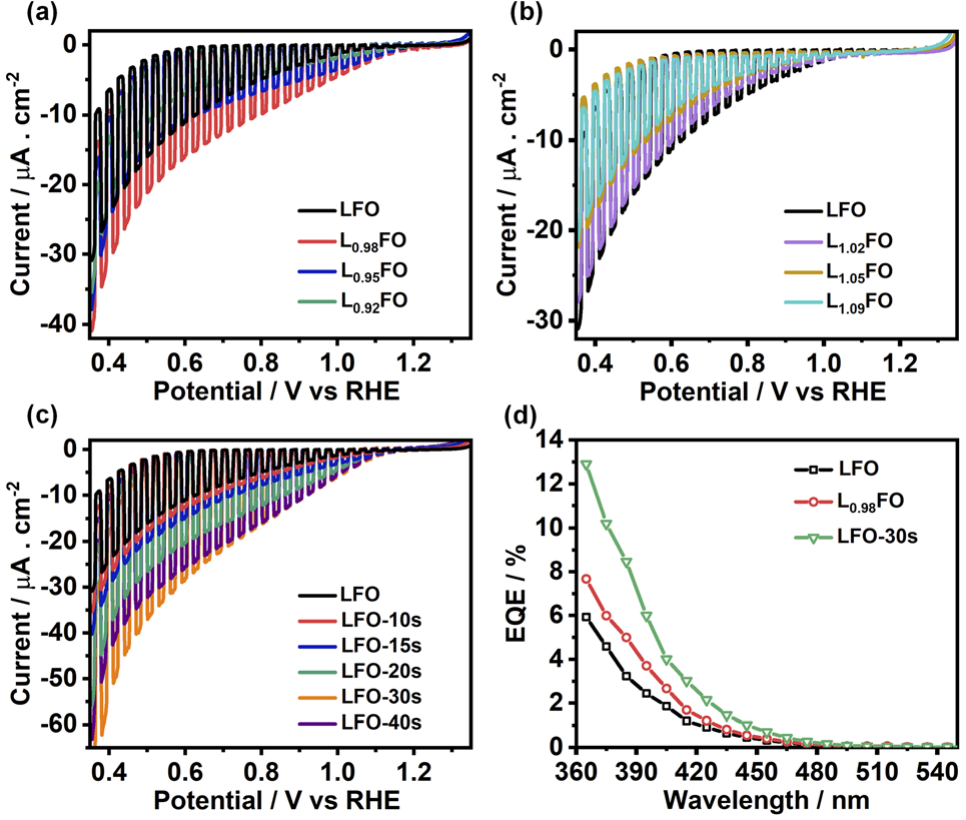
Photoelectrochemical responses associated with the oxygen reduction reaction (ORR) in O_2_-saturated 0.1 M Na_2_SO_4_ aqueous solution at pH 12 under chopped 405 nm illumination with a photon flux of 5.42 × 10^19^ m^−2^ s^−1^: effect of the bulk cation ratio (a, b) and etching time of LFO films (c). External quantum efficiency spectra obtained at 0.5 V *vs.* RHE (d), comparing the responses of LFO with the most active cation ratio and acid-treated films.

Fig. 7a and b show linear sweep voltammograms (LSV) of LFO thin films with various La : Fe ratios in O_2_-saturated Na_2_SO_4_ aqueous solution at pH 12 under the chopped illumination of 405 nm light with a photon flux of 5.42 × 10^19^ m^−2^ s^−1^. The photocurrent onset potential originating from the oxygen reduction reaction (ORR) in all compositions is above 1 V *vs.* RHE, with 2% La deficient films showing the largest photocurrent value. As the La ratio increases, we see a significant decrease in the photocurrent responses (Fig. 7b), which coincides with the increase in the density of defect states near the valence band as probed by CV (Fig. 5). Indeed, transient photocurrent responses (Fig. S4†) from Fe-poor LFO films are characterised by a slow photocurrent rise and decay in the on-transient and off-transients, respectively, which is characteristic of slow majority carrier collection due to trap states.^29^ Interestingly, the photocurrent magnitude decreases at low La-content, although the response remains in-phase with the light perturbation. This observation suggests that bulk recombination increases if the La : Fe ratio is below 0.92, indicating the formation of deeper states in the oxide electronic structure. The LSV curves of LFO films after different etching times shown in Fig. 7c reveal a systematic increase in photocurrent responses with increasing acid-etching time. The external quantum efficiency (EQE) values, recorded at 0.5 V *vs.* RHE, of the acid-treated samples, are twice the value of the non-treated LFO sample with the same bulk composition (Fig. 7d). Tauc plots constructed from EQE spectra confirm the same band gap energy obtained from the optical measurements as illustrated in Fig. S5.†

**Fig. 8:**
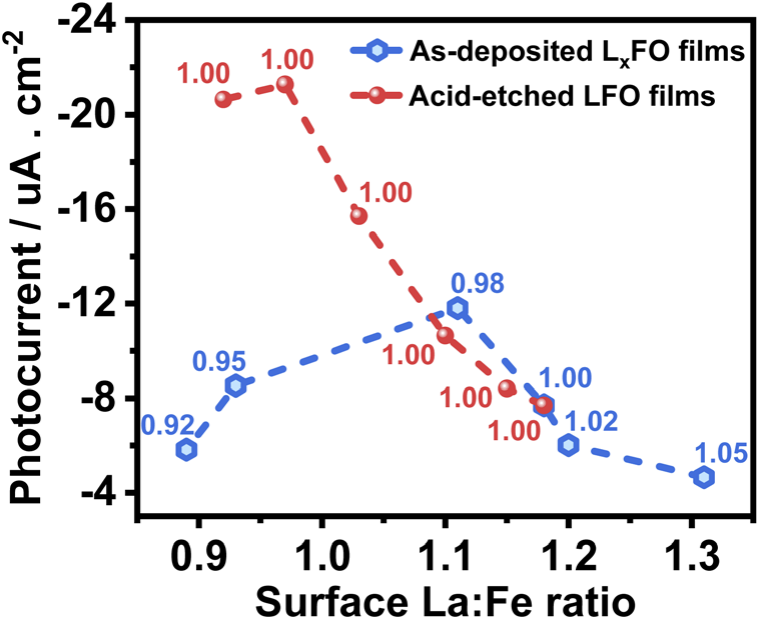
Photocurrent as a function of the surface La : Fe ratio of the LFO with various La : Fe bulk ratios (black squares) and different acid etching times of LFO films (red circles). Photoresponses were measured at 0.7 V with the same electrolyte composition and illumination level described in Fig. 6. The bulk La : Fe ratio is indicated next to each point in the plot.

Fig. 8 compiles the photocurrent responses at 0.7 V *vs.* RHE (under the same illumination conditions as in Fig. 6) as a function of the surface La : Fe ratio estimated from XPS measurements (Table S3†). The plot also features the bulk La : Fe ratio next to each of the points, which provides the first systematic decoupling of the effect of bulk and surface compositions reported for transition metal oxide photoelectrodes. The curve shows that the near stoichiometric bulk La : Fe ratio generates the maximum photocurrent responses. Furthermore, near bulk, stoichiometric films show a substantial increase in photoresponses with decreasing La content at the surface. Based on our photoemission analysis (Fig. 4), this trend suggests that increasing covalency of the Fe–O bond has a positive effect on the electron transfer dynamics to O_2_, removing surface V_Fe_ which can act as a hole trapping site.^30,31^

**Fig. 9:**
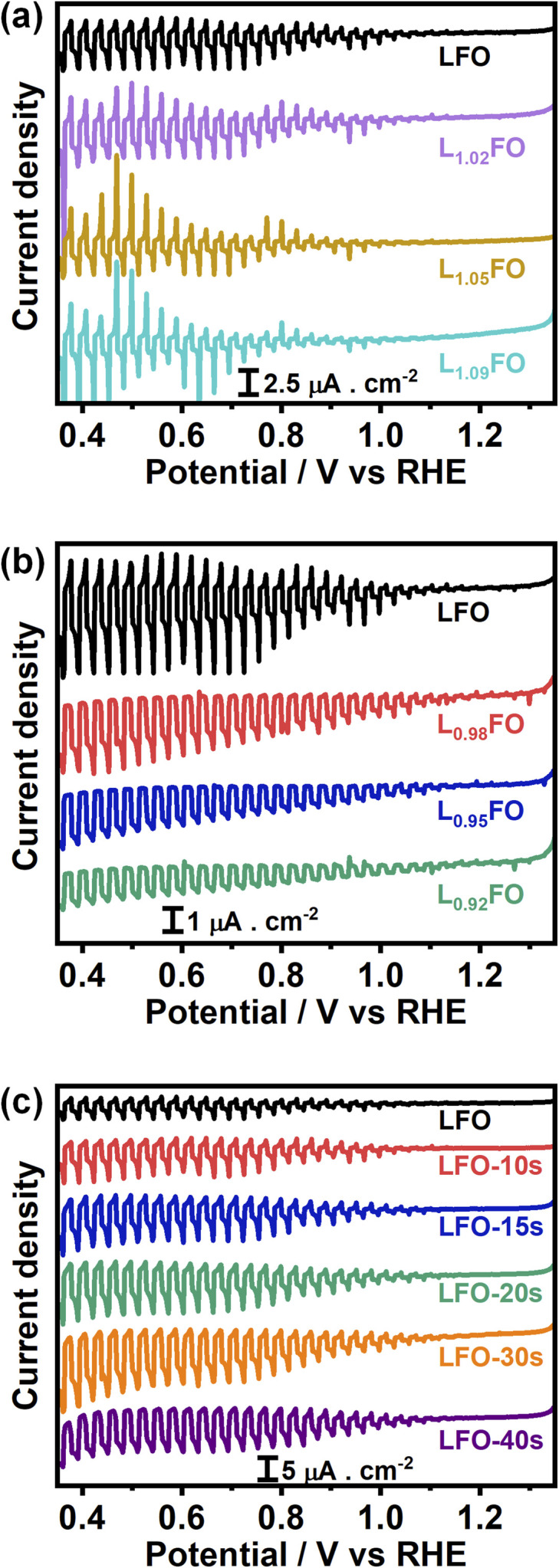
Photocurrent-potential curves in Ar-saturated 0.1 M Na_2_SO_4_ electrolyte at pH 12 with the same illumination and other experimental parameters described in Fig. 6. Thin films with different La : Fe bulk ratios (a, b) and LFO films with increasing acid-etching time (c).

Finally, Fig. 9a and b display the effect of the La : Fe bulk ratio on the photoresponses associated with the hydrogen evolution reaction (HER) in Ar-purged 0.1 M Na_2_SO_4_ electrolyte at pH 12 under the chopped illumination of 405 nm with a photon flux of 5.42 × 10^19^ m^−2^ s^−1^. La-rich films are characterised by large transient photocurrents in the on and off-transients and very small steady-state photocurrents, indicating the presence of strong surface recombination.^32,33^ The transition from La-rich to La-poor films leads to a small increase of the steady-state photocurrent, while the transient responses show a small decay upon illumination, suggesting a hindered carrier transfer and band edge unpinning.^33–35^ This behaviour provides another clear correlation between the role of V_Fe_ and carrier recombination losses. We can also see a clear and systematic increase in the photoresponses of LFO upon acid treatment. Again, this shows that surface La segregation plays a significant role in the photoelectrochemical responses of ferrite perovskites, not only due to creating a barrier for carrier transport but also due to the generation of PDs such as V_Fe_ and La_Fe_ near the surface, which act as recombination centres towards the HER reaction.

## Conclusions

For the first time, the photoelectrochemical properties of perovskite oxide thin films are systematically investigated while independently varying the bulk and surface compositions. Pure orthorhombic LFO films with a thickness of 130 nm were prepared with La : Fe ratios varying from 5% La-rich to 8% La-poor by sol–gel methods onto F : SnO_2_ electrodes. The surface La : Fe ratio of near-stoichiometric LFO was also systematically varied by acid-etching while keeping constant the bulk composition. Bulk and surface compositions were investigated by EDX, ICP-OES and XPS. XPS analysis clearly shows a linear correlation between the Fe 2p and O 1s binding energies as the surface La : Fe ratio is varied, suggesting that La deficiency leads to an increase in the Fe oxidation state and more covalent Fe–O bonding. Variation in the bulk composition shows a more complex behaviour in the case of La-rich films, which is linked to the formation of PDs such as V_Fe_. DFT calculations showed that V_La_ and V_Fe_ generate defect states near the valence band edge, which act as hole trapping sites and recombination centres, while V_O_ generates significantly deeper states.

Electrochemical studies confirm that V_Fe_ does generate states near the valence band edge energy, which manifest themselves as capacitive responses in cyclic voltammograms in the range of 0.8 to 1.4 V *vs.* RHE. As the La : Fe ratio increases, these capacitive responses increase, with a concomitant decrease of the photocurrent associated with the ORR and HER. We also observed a decrease in the photocurrent magnitude when the La bulk ratio decreases below 2%. On the other hand, variation of the surface La : Fe ratio at near-stoichiometric LFO shows a systematic increase of the photocurrent responses with decreasing La ratio. The ensemble of experimental data strongly suggests that increasing Fe–O covalency at the surface promoted by the La : Fe ratio slightly below 1 can lead to substantial improvement in photoelectrochemical responses. However, the oxygen vacancies and Fe/La antisites manifest themselves as deep states, which could be responsible for the dominant bulk recombination in these materials.

## Conflicts of interest

There are no conflicts to declare.

## Data availability

Data are available at the University of Bristol data repository, data. bris, at https://doi.org/10.5523/bris.1608ds02po8it2jufacsom4nm8.

## Author contributions

X. S. executed all the experiments reported in this Paper. D. T. performed the computational studies and contributed to the data analysis. M. L. and D. J. F. contributed to the data analysis and coordinated the project. All authors contributed to the manuscript preparation.

## Supplementary Material

SC-013-D2SC04675J-s001
