# Supplementary material for: Decoupling the impact of bulk and surface point defects on the photoelectrochemical properties of LaFeO3 thin films
Source: Chem Sci. 2022 Sep 6;13(37):11252–9. doi: 10.1039/d2sc04675j (PMC9517707; doi:10.1039/d2sc04675j)
Supplement: SC-013-D2SC04675J-s001 [file SC-013-D2SC04675J-s001.pdf]

Electronic Supplementary Material (ESI) for Chemical Science.
This journal is © The Royal Society of Chemistry 2022

Supporting Information

Decoupling the impact of bulk and surface point defects on the photoelectrochemical properties of $LaFeO_3$ thin films

Xin Sun,[a] Devendra Tiwari,[b,c] Meicheng Li,[a] and David J. Fermin[c]**

[a] State Key Laboratory of Alternate Electrical Power System with Renewable Energy Sources, School of New Energy, North China Electric Power University, Beijing, 102206, China

[b] Department of Mathematics, Physics and Electrical Engineering, Ellison Building, Northumbria University, Newcastle Upon Tyne, NE1 8ST, United Kingdom

[c] School of Chemistry, University of Bristol, Cantocks Close, Bristol BS8 1TS, United Kingdom

Corresponding authors:
E-mail: mcli@ncepu.edu.cn (M. Li)
E-mail: david.fermin@bristol.ac.uk (D. J. Fermin)

Experimental Methods

Table S1. Contrast between La:Fe ratio in the precursor solution and in the thin-film as measured by EDX and ICP-OES.

Table S2. Optimized unit cell parameters of orthorhombic $LaFeO_3$ (Pbnm) obtained from DFT calculations vs experimental data.

Table S3. Surface La:Fe ratio dependence of bulk cation ratio and acid etching time.

Table S4. Formation energies of point defects in $LaFeO_3$ (LFO).

Figure S1. XRD patterns of stoichiometric LFO and acid-etched LFO films.

Figure S2. Optical properties of LFO films with different La:Fe ratios.

Figure S3. Capacitance potential curves and Mott-Schottky plots of LFO films with different La:Fe ratios in 0.1 M Na_2SO_4 electrolyte at pH 12.

Figure S4. Transient photocurrent responses of various LFO films in O_2-saturated 0.1 M Na_2SO_4 electrolyte at pH 12.

Figure S5. Tauc plots constructed from the external quantum efficiency spectra.

Experimental Methods

Powder X-ray diffraction with Cu Ka radiation (λ = 1.54016 Å; Bruker AXS D8 ADVANCE) was used to acquire the crystal structure of various LFO films. In order to avoid interferences from the diffraction patterns of the SnO_2 substrate, LFO films were deposited onto glass slides employing the same procedure described in the Experimental and Computation Methodology section. Scanning electron microscopy (SEM, Hitachi SU8010) illustrated the top view and cross-sectional morphologies. Bulk compositions of LFO films were quantified by energy-dispersive X-ray detector (IXRF) combined with SEM as well as inductively coupled plasma−optical emission spectrometry (Varian 710−OES). X-ray photoelectron spectroscopy (ESCALAB 250Xi) revealed the surface composition information of as-prepared LFO films, including surface La:Fe ratio and elemental binding energies. The UV-Vis absorption spectra were recorded employing a Shimadzu UV-2600 spectrophotometer.

Photoelectrochemical studies were conducted using an Ivium potentiostat, based on three-electrode system (working electrode: LFO thin films coated onto FTO substrates, counter electrode: carbon rod, and reference electrode: KCl-saturated Ag/AgCl). All potentials have been converted to against the reversible hydrogen electrode (RHE). 0.1M Na_2SO_4 aqueous solutions was used as electrolyte and the pH was adjusted to 12 by adding concentrated NaOH solution. Ar-saturated or O_2-saturated electrolytes were obtained by bubbling Ar or O_2 gas (99.99%) for 20 min before starting the photoelectrochemical measurements. The solutions were gently bubbled during the experiments and more vigorous bubbling was introduced for 5 min after each measurement. Linear sweep voltammograms were measured at 5 mV s^{-1} from positive to negative potentials. Transient photocurrent measurements were performed under illumination from a 405 nm light-emitting diode (LED) with a 3.33 Hz square wave profile. The external quantum efficiency spectra were performed by a 300 W xenon lamp coupled with a monochromator in O_2-saturated 0.1M Na_2SO_4 aqueous solutions (pH 12) at 0.5 V vs RHE. The photon flux generated by the LED and lamp was measured by a calibrated Si photodiode.

Table S1. Contrast between La:Fe ratio in the precursor solution and in the thin-film as measured by EDX and ICP-OES.

	$L_{1.05}FO$	$L_{1.02}FO$	LFO	$L_{0.98}FO$	$L_{0.95}FO$	$L_{0.92}FO$	LFO-30s
**La:Fe ratio measured by EDX**	1.1	1.03	0.96	0.92	0.89	0.85	0.95
**La:Fe ratio measured by ICP-OES**	1.057	1.018	0.993	0.971	0.934	0.922	0.981

Table S2. Optimized unit cell parameters of orthorhombic $LaFeO_3$ (Pbnm) obtained from DFT calculations vs experimental data.

Lattice	Calculated	Experimental	difference / %
**a / Å**	5.5228	5.5539	-0.56
**b / Å**	7.9126	7.8535	-0.47
**c / Å**	5.5390	5.5651	0.75

Table S3. Surface La:Fe ratio as a function of the bulk ratio in L_xFO thin-films and acid etching time of LFO films ($x = 1$).

As-deposited L_xFO	$L_{1.05}FO$	$L_{1.02}FO$	LFO	$L_{0.98}FO$	$L_{0.95}FO$	$L_{0.92}FO$
**Surface La:Fe ratio**	1.31	1.2	1.18	1.11	0.93	0.89
**Acid-etched LFO-y**	**LFO**	**LFO-10s**	**LFO-15s**	**LFO-20s**	**LFO-30s**	**LFO-40s**
**Surface La:Fe ratio**	1.18	1.15	1.1	1.03	0.97	0.92

x is bulk La:Fe ratio, y is acid etching time

Table S4. Formation energies (in eV) of point defects in $LaFeO_3$.

Charge state	-3	-2	-1	0	+1	+2
Type of defect						
V_{La}	0.82	1.72	2.65	3.79		
V_{Fe}	1.38	1.71	2.41	3.24		
V_{O1}				2.87	2.11	1.69
V_{O2}				2.73	2.02	1.42
Fe_{La}			1.17	1.43	2.62	
La_{Fe}			4.13	2.95	3.58	

Native point defects are calculated using a 360-atom 3×2×3 supercell to minimize any spurious defect-defect interactions. The supercell dimensions were kept fixed, and only the internal coordinates were relaxed during calculations. The formation energy, $\Delta H_f^{D,q}$, of a defect, D, in charge state, q, are calculated using equation S1,

$$\Delta H_f^{D,q} = \left(E^{D,q} - E^{host}\right) + \Sigma\left\{n_i\left(E_i + \mu_i\right)\right\} + q\left(E_{VBM} + E_F\right) + E_{finite-size\ corr.}$$

Equation. S1

where, E^{host} is the energy of the host supercell, $E^{D,q}$ is the energy of supercell with a defect, D with a charge state, q. E_i, μ_i and n_i correspond to the reference energy in its standard state, chemical potential, and the number of atoms, i. E_{VBM} is the energy of the valence band maximum and E_F is the Fermi energy relative to E_{VBM}. $E_{finite-size\ corr.}$ is the correction to account for the limitations of such supercell defect calculations including defect-defect Coulombic interactions, spurious electrostatic potential alignment of supercell with respected to the stoichiometric supercell, and total energy due to erroneous band filling.

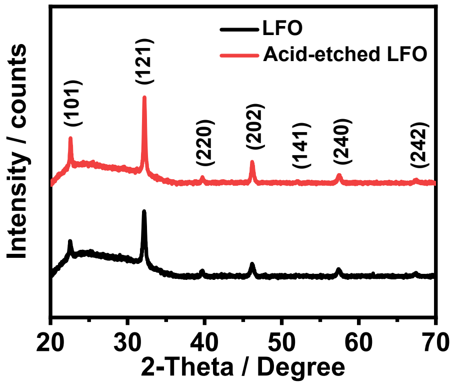

Fig. S1. XRD of as-deposited LFO before (black) and after (red) acid etching.

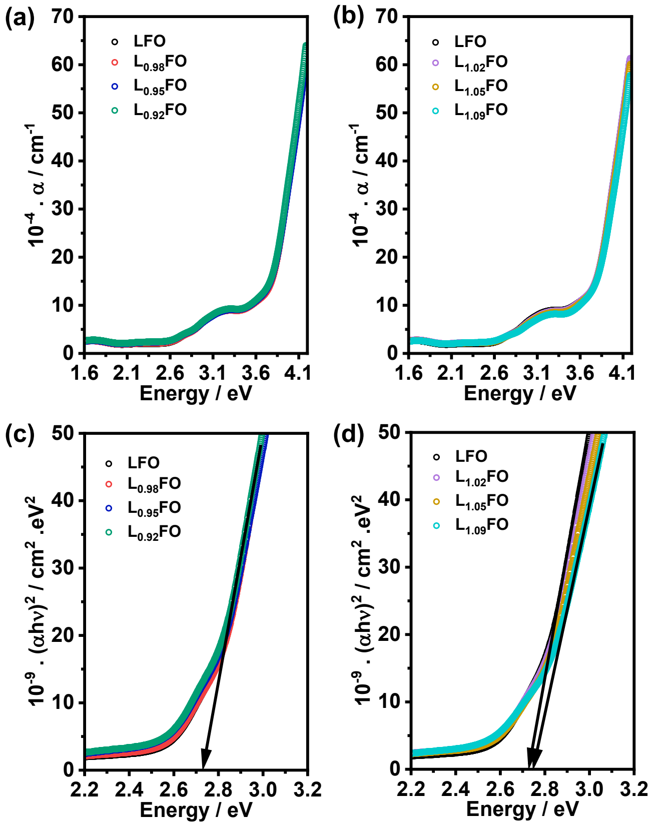

Fig. S2. Absorption constant *a* (a,b) and corresponding Tauc plots (c, d) of 130 nm LFO films with various La:Fe ratios. The optical responses and band gap (2.7 eV) show very little dependent on the bulk composition.

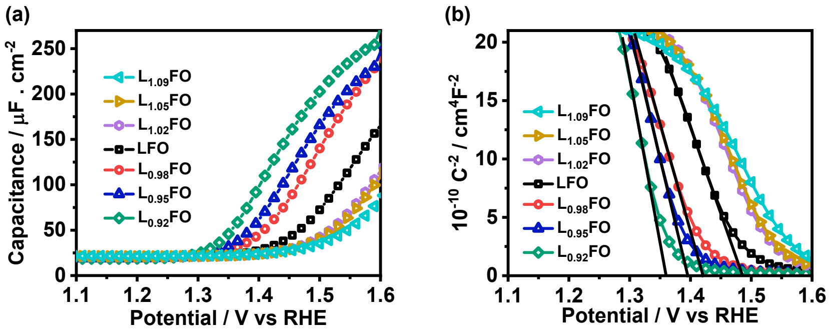

Fig. S3. Capacitance potential curves (a) and Mott-Schottky plots (b) of various L_xFO thin films in 0.1 M Na_2SO_4 aqueous solution at pH 12.

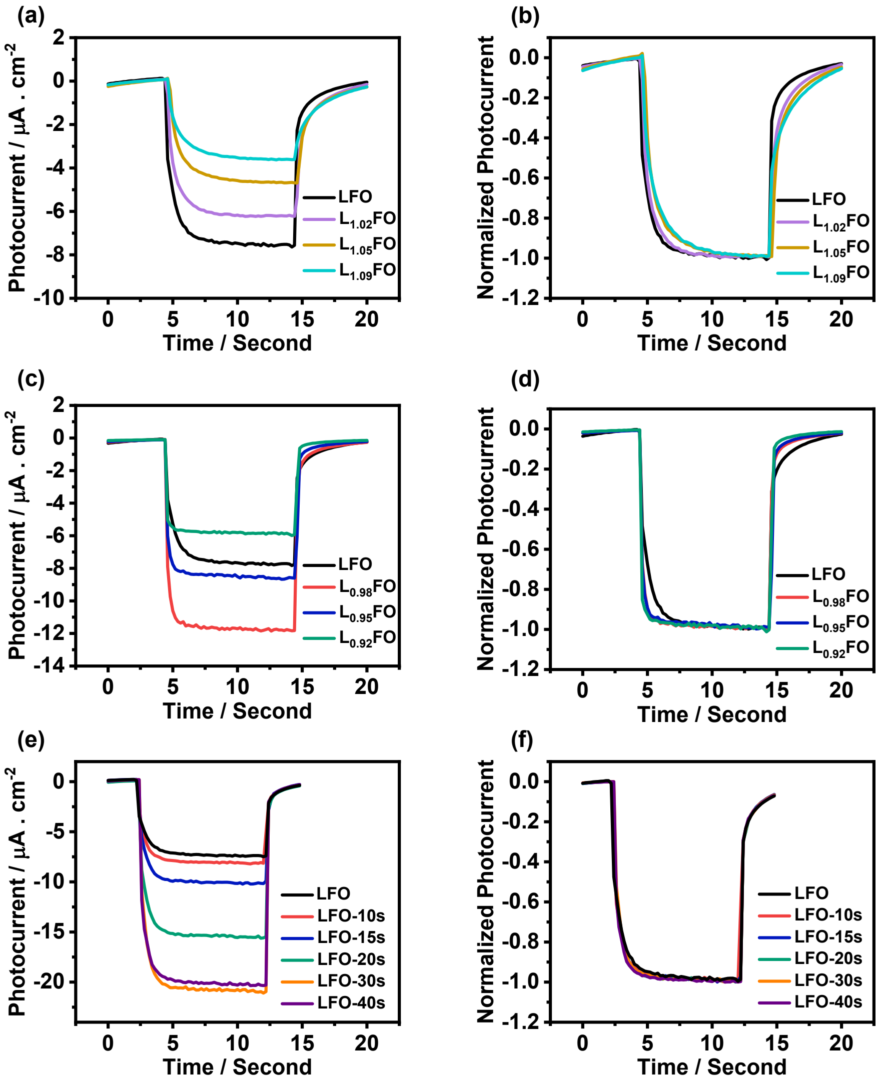

Fig. S4. Transient photocurrent responses at 0.7 V vs RHE recorded in O_2-saturated 0.1 M Na_2SO_4 electrolyte at pH 12 under the 405 nm (photon flux of 5.42×10^{19} m^{-2} s^{-1}) obtained in the case of La-rich (a), La-poor (c) and LFO after various acid etching time (e). The corresponding photocurrent transient normalized by the quasi-stationary value (b, d and f) illustrates the dynamics of on- and off-transients decoupled from the overall photocurrent magnitude.

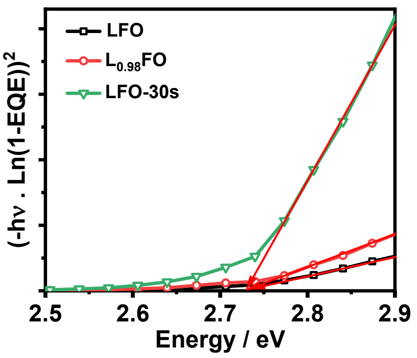

Fig. S5. Tauc representations of the external quantum efficiency spectra. The experimental conditions are described in the caption of Fig. 7.
